# *Toxoplasma gondii* ROP16 kinase silences the *cyclin B1* gene promoter by hijacking host cell UHRF1-dependent epigenetic pathways

**DOI:** 10.1007/s00018-019-03267-2

**Published:** 2019-09-06

**Authors:** Marcela Sabou, Cécile Doderer-Lang, Caroline Leyer, Ana Konjic, Sophie Kubina, Sarah Lennon, Olivier Rohr, Stéphane Viville, Sarah Cianférani, Ermanno Candolfi, Alexander W. Pfaff, Julie Brunet

**Affiliations:** 1 Institut de Parasitologie et de Pathologie Tropicale de Strasbourg, « Dynamics of Host–Pathogen Interactions » EA 7292, Fédération de Médecine Translationelle Université de Strasbourg, Strasbourg, France; 2 Service de Parasitologie et Mycologie Médicale, Hôpitaux Universitaires de Strasbourg, Centre National de Référence de la Toxoplasmose, Pôle Sérologie, Strasbourg, France; 3 Laboratoire de Spectrométrie de Masse BioOrganique (LSMBO), Université de Strasbourg, IPHC, CNRS, UMR7178, Strasbourg, France

**Keywords:** *Toxoplasma gondii*, UHFR1, ROP16, Cyclin B1, DNMT, Epigenetic regulation

## Abstract

Toxoplasmosis, caused by the apicomplexan parasite *Toxoplasma gondii,* is one of the most common infections in the world due to the lifelong persistence of this parasite in a latent stage. This parasite hijacks host signaling pathways through epigenetic mechanisms which converge on key nuclear proteins. Here, we report a new parasite persistence strategy involving *T.* *gondii* rhoptry protein ROP16 secreted early during invasion, which targets the transcription factor UHRF1 (ubiquitin-like containing PHD and RING fingers domain 1), and leads to host cell cycle arrest. This is mediated by DNMT activity and chromatin remodeling at the *cyclin B1* gene promoter through recruitment of phosphorylated UHRF1 associated with a repressive multienzymatic protein complex. This leads to deacetylation and methylation of histone H3 surrounding the *cyclin B1* promoter to epigenetically silence its transcriptional activity. Moreover, *T.* *gondii* infection causes DNA hypermethylation in its host cell, by upregulation of DNMTs. ROP16 is already known to activate and phosphorylate protective immunity transcription factors such as STAT 3/6/5 and modulate host signaling pathways in a strain-dependent manner. Like in the case of STAT6, the strain-dependent effects of ROP16 on UHRF1 are dependent on a single amino-acid polymorphism in ROP16. This study demonstrates that *Toxoplasma* hijacks a new epigenetic initiator, UHRF1, through an early event initiated by the ROP16 parasite kinase.

**Electronic supplementary material:**

The online version of this article (10.1007/s00018-019-03267-2) contains supplementary material, which is available to authorized users.

## Introduction

Toxoplasmosis, caused by the intracellular apicomplexan parasite *Toxoplasma gondii* is one of the most common infections in the world, leading to a lifelong latent infection due to the persistence of the parasites within cysts in specific host organs like the brain, the muscles or the eye [1]. While generally asymptomatic, the infection can be severe in case of primary infection during pregnancy or reactivation in immunosuppressed individuals. There is neither efficient treatment against the latent parasite forms nor a vaccine.

A particularity of *T. gondii* is the existence of numerous strains with different geographical distribution and strikingly different virulence. Originally, three major lineages had been described in Europe and North America, with type I strains being highly virulent in mouse infections, while type II and III strains showed moderate virulence [2]. More recently, genetic examination of 958 strains collected from around the world classified them into 15 haplogroups within 6 major clades [3].

To achieve intracellular persistence, *T. gondii* modulates and hijacks host cell pathways involved in various processes such as inflammation, apoptosis, metabolism, and cell growth [4]. This prevents infected cells from apoptosis and subverts the host’s immune system [5, 6]. The parasite manipulates the host cell signaling pathways by secreting kinases and phosphatases, of which some infiltrate the host cell nucleus and modulate gene expression activity. ROP16, a *T. gondii* protein kinase secreted during early stages of invasion, has been described to activate and phosphorylate immune-related transcription factors such as STAT 3/5/6 and modulate host signaling pathways in a strain-dependent manner [7–10]. Our recent results in a mouse model of ocular toxoplasmosis showed the capacity of ROP16 to influence cytokine expression and parasite control in vivo [11], demonstrating its biological importance and need to elucidate its effects on a cellular level. *Toxoplasma* strain-dependent effects on host factors have also been reported for ROP18, GRA15 and other effectors [8, 12]. For STAT3, the strain dependence of ROP16 action is determined by a single amino-acid polymorphism [13].

These changes in host genome activity imply modifying the epigenome of the host cell [14]. Until now, only few *Toxoplasma* proteins have been described as able to trigger the initial epigenetic signal: the rhoptry protein ROP18, as well as the dense granule proteins GRA15, GRA16 and probably GRA24 [4, 15–17]. *Toxoplasma* is able to imprint epigenetic marks on the host cell genome. Thus, the parasite prevents histone H3 phosphorylation and acetylation at the *TNFA* and *IL10* promoters [18, 19]. Similarly, severely impaired histone acetylation at IFN-γ-regulated promoters during infection, and a parasite-mediated defect in the recruitment of chromatin remodeling complexes have been reported [14, 20]. Decreased promoter methylation, through DNA methyltransferase activity has been shown to increase gene expression of neurons in *Toxoplasma*-infected rats [21].

CpG methylation by the DNA methyltransferases, DNMT1 and DNMT3a/b, and histone modifications are two major components of the epigenetic regulation. Transition from an active to an inactive state of transcription requires a series of coordinated modifications on histone residues. They are catalyzed by specific enzymes, but even their intrinsic affinity for their substrates is insufficient to explain their high site specificity in inducing chromatin modifications. UHRF1 (ubiquitin-like containing PHD and RING finger domain 1) plays a major role in the reading and inheritance of epigenetic code due to its ability to recruit effectors catalyzing these marks [22–25]. UHRF1 is an important epigenetic regulator with different structural domains: an N-terminal ubiquitin-like (UbL) domain, a tandem tudor (TTD) domain, a plant homeodomain (PHD), a SET and RING (really interesting new gene)-associated (SRA) domain. These domains are associated with binding to methylated DNA (SRA domain), histones (TTD and PHD domains) and epigenetic effectors (SRA, TTD, PHD domains) [24]. UHRF1 and the associated proteins play a central role in epigenetic modifications such as DNA methylation and histone modifications. Indeed, UHRF1 is associated with maintenance of DNA methylation patterns by recruiting DNMT1 to hemi-methylated CpG, and histone modifications by interacting with HDAC1 and DNMT1 [23, 25, 26]. UHRF1 coordinates epigenetic silencing of tumor suppressor genes and plays a key role in cell cycle, epigenetic regulation in the development and progression of cancers and metastasis [24, 27].

We have previously shown that both cyclin B1 mRNA and protein are downregulated in *T. gondii*-infected cells, resulting in dissociation of the cyclin B1/Cdk1 complex, which is necessary for the G2/M transition. We also found that infection-induced upregulation of UHRF1 expression is responsible for the host cell arrest at the G2 phase and is essential for parasite proliferation [28]. ROP16 was reported to interact with p53 resulting in G1 cell cycle arrest [29].

Here, we report a new parasite strategy involving ROP16 which targets UHRF1. This event prevents chromatin remodeling at the *cyclin B1* gene (*CCNB1*) promoter through recruitment of phosphorylated UHRF1 associated to a repressive multienzymatic complex. This leads to deacetylation and methylation of histone H3 surrounding the *CCNB1* promoter to epigenetically silence its transcriptional activity. Moreover, *T.* *gondii* infection causes DNA hypermethylation in its host cell by upregulation of DNMTs. This study demonstrates that *Toxoplasma* hijacks another epigenetic initiator, UHRF1, through an early event initiated by the ROP16 kinase.

## Materials and methods

### Cells and parasites

The human trophoblast cell line BeWo was obtained from the American Type Culture Collection (Mannassas, VA) and cultured in FK12 medium (Fisher Scientific, Illkirch-Graffenstaden, France), supplemented with 10% heat inactivated FCS (Invitrogen, Cergy Pontoise, France), 10 U/ml penicillin, 10 µg/ml streptomycin (Invitrogen). The human astrocytic cell line U-118MG was cultured in DMEM medium (Fisher Scientific), supplemented as above plus 2 mM glutamine. Cell cultures were kept at 37 °C, 5% CO2 and cell numbers were determined with a Neubauer cell counting chamber using a Trypan blue (Corning, Corning NY) exclusion test.

The virulent RH *T. gondii* strain was originally obtained from the French Biological Resource Center *Toxoplasma* (CRB *Toxoplasma*; Laboratoire de Parasitologie, CHU Reims, France). The RHΔROP16 strain was kindly provided by J. Boothroyd, Stanford University. Preliminary assays showed similar parasite proliferation and UHRF1 regulation of the RH and the RHΔKU80 strain, which was used to create the KO strain. RH parasites were thus used as control strain. Tachyzoites were maintained in human THP1 monocyte cultures or by weekly passages in Swiss Webster mice. Before use, they were washed twice in PBS and counted using Trypan blue exclusion test.

To create ROP16 mutant plasmids, the coding region of ROP 16 was amplified by PCR on genomic DNA from the RH, PRU and LEF strains using primers 5′-CGGAATTCATGAAAGTGACCACGA-3′ and 5′-CGCTCTAGACTACATCCGATGTGAAG -3′ including the *Eco*RI and BglII restriction sites. An amplified fragment of 2124pb was purified and inserted into the cloning vector pGEMT^®^ (Promega) by ligation after A-tailing. PGEMT-ROP 16 was digested by EcoRI and BglII restriction enzymes and cloned in pCDNA3-HA vector (Invitrogen).

The mutant plasmid ROP16ΔCat, deficient for the kinase catalytic domain was generated using the a site-directed mutagenesis kit (Stratagene, San Diego, USA) on the expression vectors containing the wild-type gene of ROP16 The primers used were sense 5′-GAGTGCCGATCGCTCAGCAAGCCCCTGG-3′ and antisense 5′-CCAGGGGCTTGCTGAGCGATCGGCACTC-3′. The mutants L503S for RH and LEF strains and S503L for PRU were likewise generated, using the primers (L503S) sense 5′ CCA TTA ATT GAT GGC TCC GCA TCG AAC AGT CTA GTC CAG TC 3′; antisense 5′ GAC TGG ACT AGA CTG TTC GAT GCG GAG CCA TCA ATT AAT GG 3′ and (S503L) sense 5′ CCA TTA ATT GAT GGC TCC CCA TTG AAC AGT CTA GTC CAG TC 3′; antisense 5′ GAC TGG ACT AGA CTG TTC AAT GGG GAG CCA TCA ATT AAT GG 3′.

### Transfection of host cells and luciferase assays

The corresponding nucleotides of UHRF1 were cloned into a siRNA expression vector, psiU6BX3, constructed as previously described [30]. A EGFP-siRNA or UHRF1-siRNA (si-3: 2123–2141) plasmid construct was transfected to cells for 24 h and incubated with 900 µg/ml of G418 for a further 48 h before infection with *T.* *gondii* as indicated in the figure legends. Cells were then harvested for Western blotting. pGL3-UHRF1-Luc plasmids were generously donated by M. Unoki, University of Tokyo, Japan. BeWo cells were transfected with plasmids (2 µg/well) for 24 h with X-tremeGENE™ (Sigma-Aldrich, Saint-Quentin-Fallavier, France) transfection reagent and infected with *T.* *gondii* for the indicated times and harvested for luciferase activity measurements with 250 µL of Bright-Glo luciferase assay Buffer (Promega, Charbonnières-les-Bains, France). Results are expressed as a ratio of the protein quantification, as our preliminary tests showed that the protein masses of the parasites are negligible compared to the cell protein contents.

### Chemicals and antibodies

Polyclonal anti-*T. gondii* antibody was raised in New Zealand rabbits by several injections of 50 µg of soluble *T. gondii* antigen suspended in Freund’s incomplete adjuvant. The IgG fraction of this serum was purified by chromatography on DEAE Trisacryl (l M) and tested by ELISA. The mouse monoclonal antibody against UHRF1 (clone 1RC1C-10) was engineered as described elsewhere [28]. The anti-cyclin B1, anti-p-Ser and anti-actin mouse monoclonal antibodies or anti-DNMT1 rabbit polyclonal antibody were obtained from Santa Cruz Biotechnology (Santa Cruz, CA).

Alexa Fluor 488 goat anti-rabbit IgG was obtained from Invitrogen (Carlsbad, CA).

Goat F(ab’)2 fragment anti-mouse IgG-peroxidase, donkey F(ab’)2 fragment anti-rabbit IgG-peroxidase, Tween-20 and the protease inhibitor cocktail were purchased from Roche Diagnostics (Basel, Switzerland). G 418 was purchased from Sigma-Aldrich (Saint-Louis, MO).

The ECL detection system was obtained from Amersham Biosciences (GE Healthcare Europe GmbH, Orsay, France). TriReagent was purchased from Molecular Research Center (Cincinnati, OH).

### Flow cytometry

BeWo cells were grown in six-well plates (Dutscher, Brumath, France) and infected at sub-confluent conditions with *T.* *gondii* for the indicated times at 37 °C. The cells were harvested by trypsinization (Fisher Scientific) and gently washed three times with 1 ml of PBS. Cell suspensions were fixed by incubation for 15 min on ice in 0.4 ml of 5% formaldehyde in PBS (v/v), washed with PBS; then resuspended in 1 ml of absolute ethanol and stored at − 20 °C until use. Infected cells were identified by flow cytometry. Briefly, cells were washed and stained for 45 min with rabbit anti-*T.* *gondii* IgG antibody. Then, samples were washed and incubated with Alexa Fluor 488 goat anti-rabbit for 45 min and washed again. DNA labeling was obtained by incubation for 2 h with 50 μg/ml of propidium iodide and 50 μg/ml of RNAse A (Euromedex, Souffelweyersheim, France) at room temperature in the dark. Fluorescence was measured in a FACScan flow cytometer (Becton–Dickinson, Franklin Lakes, NJ) and analyzed with the CellQuest software package.

### Western blotting and co-immunoprecipitation

Whole cell extracts were prepared as described elsewhere [30]. Blots were probed with the indicated antibodies at 1 µg/ml. For co-immunoprecipitation of UHRF1, infected or uninfected cell extracts were incubated with protein G beads coupled to anti-UHRF1 monoclonal antibody (5 µg) in 1 ml PBS supplemented with protease inhibitors for 2 h at 4 °C. Beads were washed five times with PBS and bound proteins were removed from the beads, denatured using loading buffer and separated on 10% SDS-PAGE gels, blotted and finally probed with 1 µg/ml of antibodies. Secondary peroxidase conjugated antibodies were used at 0.16 µg/ml. Signals were visualized by chemiluminescence using the ECL detection system.

### Proteomics

Samples were separated on a 10% SDS-PAGE gel stained with Coomassie blue. After migration, each line was cut every 2 mm and the gel plugs transferred into a 96-well plate.

In-gel digestion was performed with an automated protein digestion system, a MassPrep Station (Waters, Manchester, U.K.). The gel plugs were washed twice with 50 µL of 25 mM ammonium hydrogen carbonate and 50 µL of acetonitrile. The cysteine residues were reduced by addition of 10 mM dithiothreitol at 60 °C and alkylated by addition of 50 µL of 55 mM iodoacetamide. After dehydration with acetonitrile, the proteins were cleaved in gel with a 12.5 ng/µL solution of modified porcine trypsin (Promega, Madison, WI) in ammonium hydrogen carbonate (≈ 15 µL). The digestion was performed overnight at 37 °C. Tryptic peptides were extracted twice: with 40 µL of a 60/40/0.1 ACN/H_2_O/HCOOH solution for 1 h and with 40 µL of pure ACN. The collected extracts were pulled and evaporated to 10 µL with a vacuum centrifuge (Speedvac^®^, Thermo Savant SPD111 V, Waltham, MA, USA).

For MS analysis, each sample was analyzed on a nanoAcquity™ (Waters, Manchester, U.K.) coupled to a Synapt G1 (Waters, Manchester, U.K.), which was equipped with a nanoelectrospray ion source. Peptide separation was carried out on a RP-HPLC column C18 (75 µm × 200 mm length nanoAcquity™UPLC™, porosity 1.7 µm). The gradient was performed by a mix of two solvents A (0.1% formic acid in water) and B (0.1% formic acid in acetonitrile). Separation was performed at 300 nL/min flow rate using a 35 min gradient from 10 to 40% B. The capillary, sample cone and extraction cone voltages were set to 3000 V, 35 V and 40 V, respectively. The temperature of the source was set to 90 °C.

Mass calibration was achieved using the fragmentation of glyco-fibrin peptide over the range 250–2000 Da. Acquisition was performed with the Masslynx software 4.1. (Waters, Manchester, UK). The MS survey scan was acquired on the range m/z 250–1500 with a scan time of 0.5 s. The five most intense ions of the MS spectra were selected for MS/MS (intensity threshold 20 counts). CID fragmentation was performed using argon as collision gas and with collision energy profile optimized for various mass ranges of precursor ions. The scan range for MS/MS acquisition was from m/z 50 to 2000 Da with a scan time of 0.8 s. If the intensity of the MS/MS was less than 4500 cps, the MS/MS scan lasted 2.3 s. A selected ion is excluded for 4 s after the selection.

Data collected during a nanoLC-MS/MS analysis were automatically processed and converted into a.pkl file using ProteinLynx Browser 2.3 (Waters, Manchester, U.K.). These files were then submitted to Mascot (Matrix Science, London, U.K.) against a mixed human (Swiss prot, 23/02/2012) and toxo (Toxodb, vr6.3, 22/01/2011) decoy database (88 468 entries). Searches were performed with a tolerance on mass measurement of 15 ppm for the precursor and 0.07 Da on the fragments. Carbamidomethylation of cysteine residues and oxidation of methionine residues were searched as variable modifications. Up to one missed cleavage was allowed.

Scaffold 3.00.03 (Proteome Science, Portland, Oregon) was used for identification validation and false positive rate estimation for protein identification. Selection filters were applied to obtain a false positive rate (FDR) less than 1%. Seven proteins of interest were selected, MS/MS spectra extracted and manually verified. These seven proteins of interest were then validated by immunoprecipitation.

### Quantitative PCR and RT-PCR analysis

Cells were cultured in six-well plates, infected with *T.* *gondii* for the indicated times and lysed with TriReagent. Total RNA was extracted according to the manufacturer’s recommendations. 5 µg of RNA were then reverse transcribed using qScript cDNA Synthesis Kit (Quantabio). Primers were used at a final concentration of 0.5 µM: *glyceraldehyde*-*3*-*phosphate dehydrogenase* (*GAPDH*, 137 bp) sense 5′-AGC AAT GCC TCC TGC ACC ACC AAC-3′; antisense 5′-CCG GAG GGG CCA TCC ACA GTCT-3′; *DNMT1* (144 bp) sense 5′- AGGACAGGGGACCCACGAAA-3′; antisense 5′- ACA CCT CAC AGA CGC CAC AT-3′; *UHRF1* (240 bp) sense 5′- GGG GCT ATG AGG ATG ATG TG-3′; antisense 5′-TCT TGC CAC CCT TGA CAT T-3′. Quantification of transcripts was performed by an external standard curve. Real-time PCR was carried out on the CFX Connect™ Real-Time PCR detection system (Bio-Rad, Marnes la Coquette, France) using the SsoAdvanced™ Universal SYBR^®^ Green Mix (Bio-Rad). After 30 s of denaturation at 95 °C, the reaction was cycled 40 times, 10 s at 95 °C, 10 s at 58 °C and 30 s at 72 °C. Product specificity was determined by melting curve analysis.

### Chromatin immunoprecipitation (ChIP) analysis

Cells were cultured in 10 cm dishes and then infected with *T.* *gondii* for the indicated times. ChIP was performed according to manufacturer’s recommendations (ChIP-IT Express enzymatic kit, Active Motif, CA, USA). The supernatant containing the sheared chromatin was incubated with anti-UHRF1 or anti-Actin (as negative control) antibody and magnetic beads overnight at 4 °C on a roller shaker. The beads were washed and incubated with 100 µl of elution buffer at 65 °C for 2.5 h. Cross-linking was reversed by a 1.5 h incubation with 2 µl of proteinase K at 37 °C. DNA was extracted using the QiaMini Kit (Qiagen). DNA samples from the ChIP experiments were subjected to PCR using *CYCLINB1* promoter-specific primers (sense 5′-CGC CAA TGG GAA GGG AGT-3′, antisense 5′-CCA CAA GAC GAA GAG GGG C-3′) and TaqDNA polymerase (Invitrogen). The reaction contents were heated to 94 °C for 15 min for polymerase activation followed by 35 cycles at 94 °C for 30 s, 55 °C for 30 s, 72 °C for 30 s and a final step at 72 °C for 7 min.

### Methylation analysis

BeWo cells were cultured and infected in six-well plates, as described above. DNA was extracted using QiaMini Kit (Qiagen). Bisulfite conversion was performed using MethylDetector (Active Motif), according to the manufacturer’s recommendations. The modified DNA was then subjected to PCR amplifications of the repetitive LINE1 sequence, using either primers specific for the methylated sequence (sense 5′-AAG ATG GTC GAA TAG GAA TAG-3′, antisense 5′-CAC TCC CTA ATA AAA TAA ACC-3′) or for the unmethylated sequence (sense 5′-AGA TGG TTG AAT AGG AAT AGT-3′, antisense 5′-CAC TCC CTA ATA AAA TAA ACC-3′), at an annealing temperature of 55 °C. Cycle numbers were optimized to stop the reaction in the logarithmic phase of amplification. Quantity One 4.6.5 software (Bio-Rad, Philadelphia, PA) was used for measuring the density of PCR bands.

### DNMT assay

BeWo cells were cultured in six-well plates and then infected with *T.* *gondii* for the indicated times. DNMT activity was performed on 10 µg of nuclear extract using DNMT activity (Active Motif), according to manufacturer’s recommendations. Absorbances were read at 450 nm. As *T. gondii* was shown not to possess DNMT activity [31], these results fully represent host cell DNMT activity.

### Statistical analysis

Statistical analysis was performed using Student’s *t* test for comparisons between two groups, or one-way ANOVA followed by Dunnett’s post-test for comparisons relative to a control group. All tests were performed using GraphPad. A *P* value below 0.05 was considered statistically significant.

## Results

### Activation of UHRF1 promoter and phosphorylation of UHRF1 in *T.* *gondii*-infected cells correlate with rhoptry secretion

**Fig. 1:**
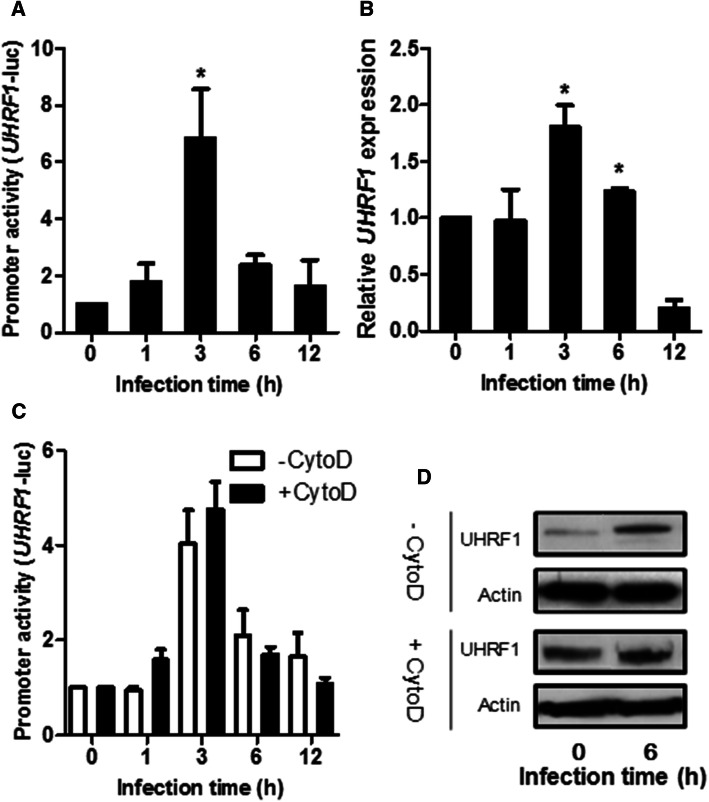
*T. gondii* infection activates UHRF1 promoter activity and phosphorylates UHRF1. **a** BeWo cells were transfected with UHRF1-promoter-luciferase reporter plasmid for 24 h, then infected for the indicated times with *T.* *gondii* (RH strain) at a ratio of 1:1 and assessed for luciferase activity (mean ± SEM of three separate experiments performed in triplicate). ^*^P < 0.05, compared to control. **b** BeWo cells were infected for the indicated times. UHRF1 mRNA was quantified by reverse transcription and real-time PCR (mean ± S.E.M. of three separate experiments performed in triplicate). ^*^P < 0.05, compared to control. **c** UHRF1 promoter activity in BeWo cells was assessed as above following infection with *T.* *gondii* pretreated or not with cytochalasin D. Mean ± SEM of three separate experiments performed in triplicate. ^*^P < 0.05, compared to control. **d** Western blot analysis of UHRF1 protein expression in BeWo cells infected with *T. gondii* pretreated or not with cytochalasin D. Actin served as a loading control. Data are representative of at least three independent experiments

We previously reported that UHRF1 expression is rapidly upregulated in *T.* *gondii*-infected trophoblastic BeWo cells [28], specialized cells of placental origin that have a central position in the control of materno-fetal passage of pathogens, including *T.* *gondii* [32]. Here, we wanted to study the kinetics of UHRF1 promoter activation and mRNA expressions upon *T.* *gondii* infection. First, host cells were transfected with an UHRF1 promoter-luciferase (UHRF1-luc) reporter plasmid, followed by infection with the virulent RH strain of *T. gondii*. Infection significantly induced UHRF1 promoter activity at 3 h post-infection (Fig. 1a). To complete this result, UHRF1 mRNA was analyzed by quantitative reverse transcription PCR. The results also show a significant increase in UHRF1 mRNA expression at 3 h post-infection (Fig. 1b). UHRF1 promoter activity did also increase in non-infected cells with culture time, but slower and not to the same extent as in infected cells (Suppl. Fig. 1).

We also examined the effect of *T. gondii* infection on *UHRF1* promoter activity and cell cycle inhibition in astrocytes, primary targets of *T. gondii* in the brain [33]. We also observed an increase of UHRF1 promoter activity, but more gradual between 0 and 12 h of infection (Suppl. Fig. 2), in contrast to the sharp early peak seen in BeWo cells, as well as a visible, but less pronounced cell cycle block.

In the early phases of *T. gondii* invasion, rhoptry proteins are released into the host cell and modulate some of its processes. Some of them present kinase activity, and are crucial in the host–pathogen interactions [10, 34]. To investigate the role of these secreted proteins, we pretreated the parasites with cytochalasin D, which allows rhoptry protein discharge into the cell, but not host cell penetration of the parasite [35]. As shown in Fig. 1c, cytochalasin D-treated *T. gondii* were still able to stimulate UHRF1 promoter activity at 3 h of infection. These data strongly suggest that *Toxoplasma*-mediated activation of the UHRF1 promoter does not need parasite penetration of the host cell, but may be promoted by rhoptry secretion into the target cells. We also looked for UHRF1 expression on the protein level following *T. gondii* infection, with or without cytochalasin D pre-treatment. Six hours post-infection, UHRF1 protein expression was increased in the presence or not of cytochalasin D. These results confirmed that the increase in UHRF1 expression is due to rhoptry secreted proteins and does not need parasite penetration.

### ROP16-dependent activation and phosphorylation of UHRF1

To further characterize the interactions between *T. gondii* proteins and UHRF1, we employed the yeast two-hybrid technique to look for candidate proteins. The sequences of the strongest confirmed, non-redundant UHRF1-interacting clones were analyzed with bioinformatic tools, including ToxoDB (ToxoDB.org release 2.3) (data not shown). Several parasite proteins were identified, of which we selected the kinase ROP16 as the most likely candidate due to its role in parasite virulence and its demonstrated catalytic activity. We hypothesized that ROP16 might increase UHRF1 promoter activity and phosphorylate UHRF1.

**Fig. 2:**
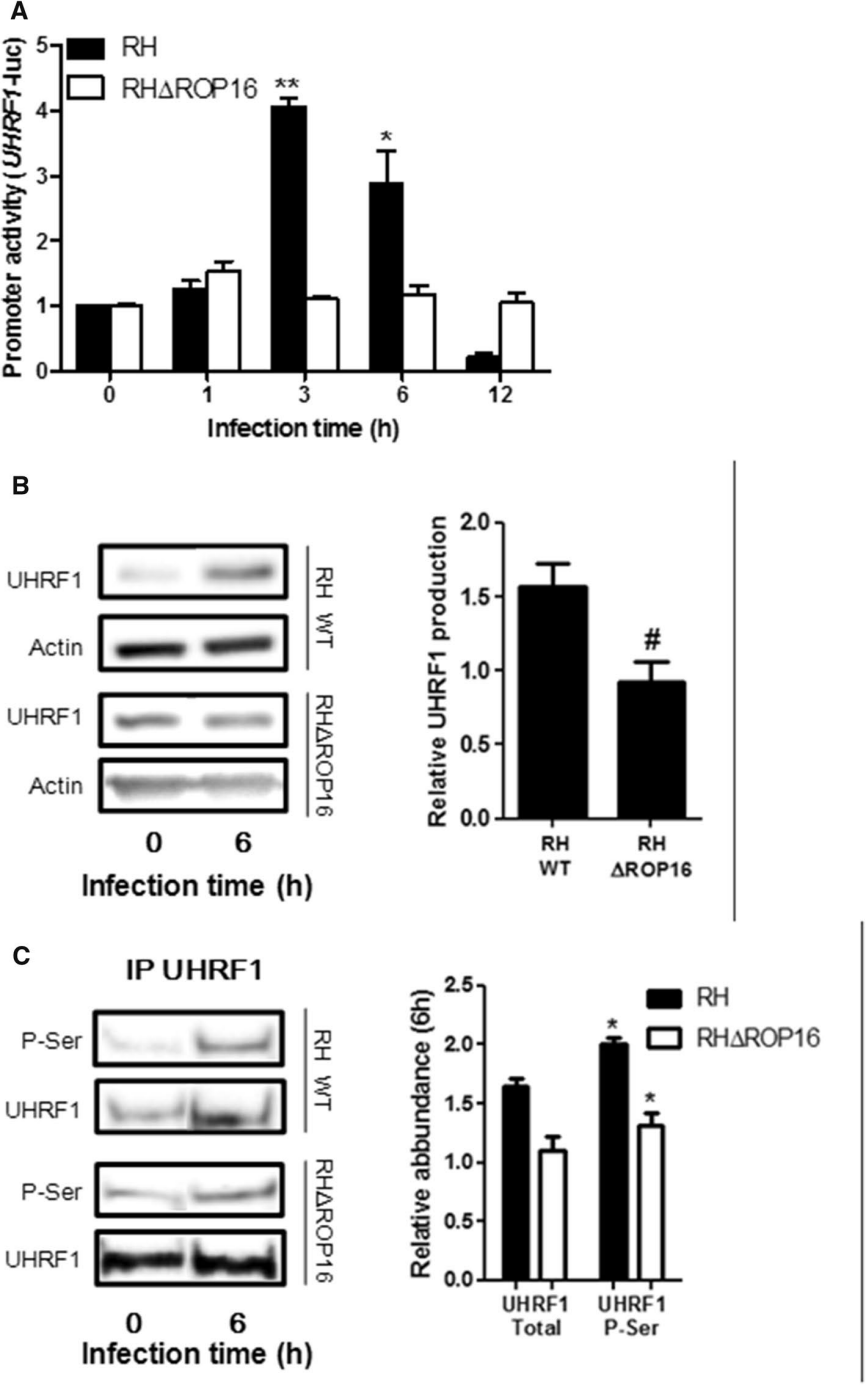
*T. gondii* ROP16 is responsible for UHRF1 activation and phosphorylation. **a** BeWo cells were transfected with UHRF1-dependent luciferase reporter plasmid for 24 h, then infected with the RH WT or RHΔROP16 strains for the indicated times. Luciferase activities were analyzed and shown as mean ± SEM of three separate experiments performed in triplicate. ^*^P < 0.05, ^**^P < 0.01, compared to 0 h. **b** Cells were infected for 6 h with RH WT or RHΔROP16 strains. Whole cell lysates were analyzed by Western blot using anti-UHRF1 antibodies. Actin served as a loading control. Data are representative of at least three independent experiments. **c** Immunoprecipitation of UHRF1 was performed on whole cell lysates of uninfected or 6 h infected cells followed by Western blot analysis of the indicated proteins. The quantitative graphs in (**b**) and (**c**) show the relative changes at 6 h of infection, compared to 0 h (mean ± SEM of three separate experiments). ^*^P < 0.05, compared to non-infected cells

To analyze if UHRF1 could be an as yet unknown substrate of the ROP16 kinase, we infected BeWo cells with an RH mutant deficient for ROP16 (RHΔROP16). Intracellular replication of RHΔROP16 parasites was similar to that of the RH WT parasites (data not shown). As shown in Fig. 2a and b, the UHRF1 promoter was not activated in cells infected with RHΔROP16 parasites, and UHRF1 protein expression was not increased in these cells, in contrast to cells infected with RH WT parasites.

We have previously shown that, contrary to its promoter activity, UHRF1 protein levels continuously increase up to 24 h post-infection [28]. Therefore, we hypothesized that *T. gondii* might induce post-translational modifications of the UHRF1 protein to stabilize it. Phosphorylation is an essential process in regulating the activity of nuclear proteins. Phosphorylation of UHRF1 increases its binding to the promoters of target genes [36]. Therefore, we examined the phosphorylation of UHRF1 by immunoprecipitation of total UHRF1 protein and subsequent detection using an antibody against pan-phosphorylated serine (P-Ser). A twofold increase of phosphorylated UHRF1 was detected upon infection with RH WT, whereas this increase was less obvious with RHΔROP16 parasites (Fig. 2c). These results suggested that ROP16 is probably not the only, but a major factor for UHRF1 phosphorylation and activation.

**Fig. 3:**
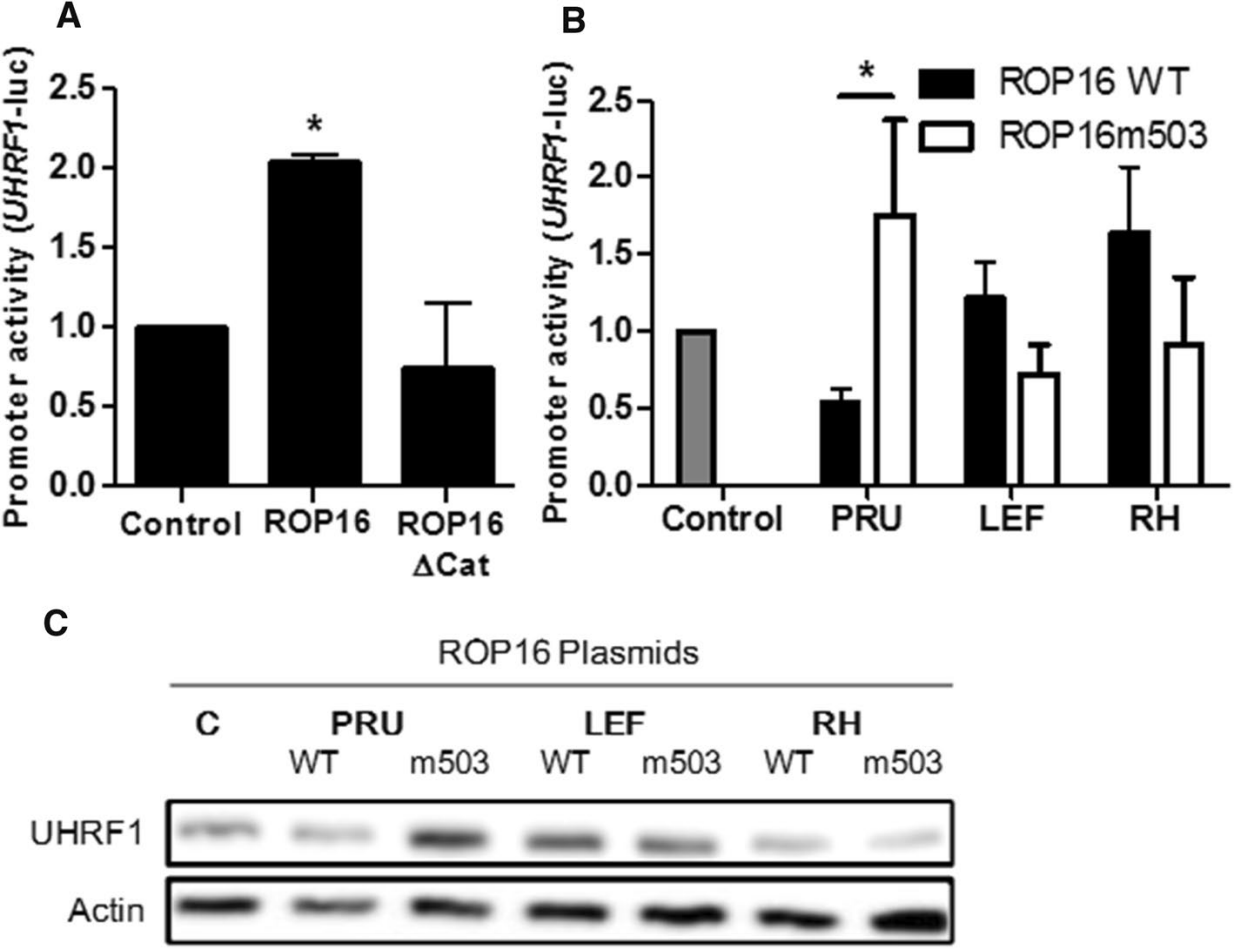
A single ROP16 amino acid determines the strain-specific activation of UHRF1. **a** BeWo cells were transfected with UHRF1-promoter-luciferase reporter plasmid for 24 h, then with ROP16 WT, ROP16 mutant plasmid without the catalytic kinase domain (ROP16ΔCat) (both from *T. gondii* RH strain) or the empty control plasmid. Luciferase activity was then analyzed and shown as mean ± SEM of three separate experiments performed in triplicate. ^*^P < 0.05, compared to control. **b** UHRF1 promoter activity in BeWo cells was assessed as above following transfection with one of the following plasmids: control plasmid (gray bar), ROP16 WT plasmids (black bars) or ROP16m503 mutant plasmids (white bars) from PRU, LEF and RH strains. Mean ± SEM of three separate experiments performed in triplicate. ^*^P < 0.05, between ROP16 WT and ROP16m503. **c** Western blot analysis of UHRF1 expression in cells transfected for 24 h with the control plasmid, ROP16 WT or ROP16m503 mutant plasmids from PRU, LEF or RH strains. Actin served as a loading control. Data are representative of at least three independent experiments

To further investigate the influence of ROP16 and its catalytic kinase domain, a plasmid containing the gene coding for a ROP16 mutant without a functional catalytic domain (ROP16ΔCat) was transfected into BeWo cells. Cells were also transfected with a plasmid containing the corresponding RH wild-type ROP16 and with an empty plasmid as control. As shown in Fig. 3a, overexpression of wild-type ROP16, but not ROP16ΔCat induced significant activation of the UHRF1 promoter.

Again, we also examined the effect of transfection of wild-type and mutant ROP16 on UHRF1 promoter activity in astrocytes We obtained similar results as in BeWo cells, with activation of UHRF1 promoter activity only upon transfection with the wild-type ROP16, but not ROP16ΔCat (Suppl. Fig. 3).

Together, these results demonstrate that ectopic expression of ROP16 is sufficient to activate UHRF1 promoter activity and that this activation depends on ROP16 kinase activity.

### A single ROP16 amino acid determines the strain-specific activation of UHRF1

The genus *Toxoplasma* consists of only one species, *T.* *gondii*, but the parasite population is extremely diverse. For ROP16, the decisive difference between virulent and avirulent alleles for STAT3 activation has been localized to the presence of leucine or serine, respectively, at position 503 [13]. To test this hypothesis on UHRF1, different types of ROP16 mutants from different strains were produced. The ROP16 503 leucine of type I (RH) and the atypical strain LEF were replaced by serine, and vice versa for the type II strain (PRU) ROP16, where serine was replaced by a leucine at the same position. As shown in Fig. 3b, RH and LEF 503 mutant plasmids induced less activation of the UHRF1 gene promoter than their wild-type counterparts, while the PRU 503 mutant protein activated the UHRF1 gene promoter significantly stronger than the PRU wild type.

We next analyzed UHRF1 in cells expressing the same WT or 503 ROP16 mutant proteins on the protein level and got similar results (Fig. 3c). Expression of the PRU 503 ROP16 mutant strongly increased UHRF1 protein levels. Taken together, these results suggest that this single locus on amino acid at 503 determines the impact of ROP16 on UHRF1 expression.

### UHRF1 is recruited to the *cyclin B1* gene promoter and suppresses its expression in *Toxoplasma*-infected cells

**Fig. 4:**
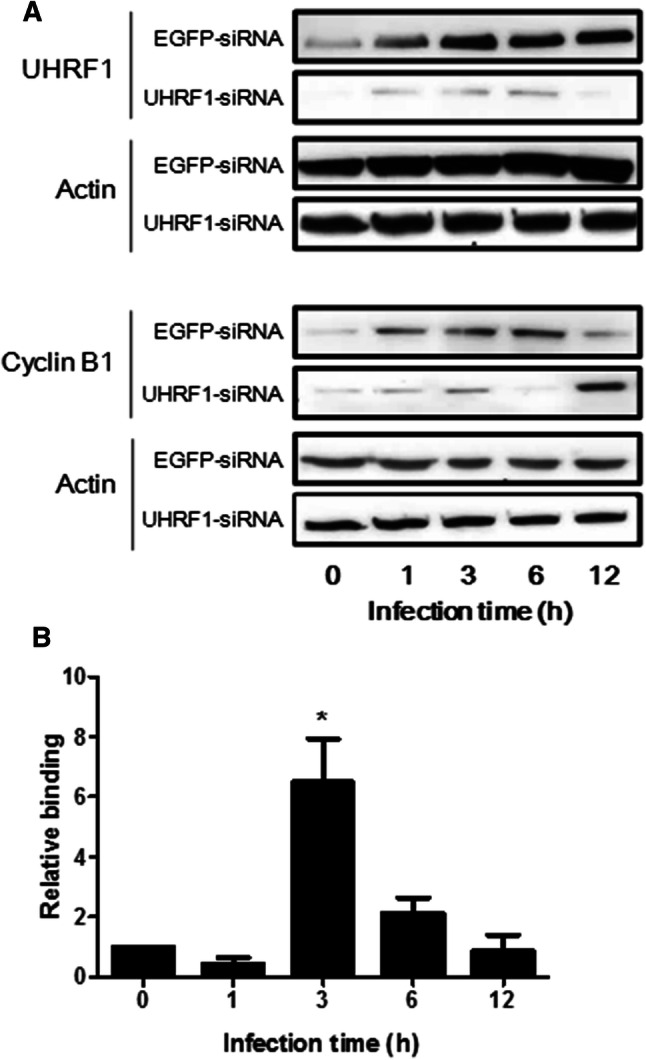
UHRF1 regulates cyclin B1 expression in *T. gondii*-infected cells. **a** BeWo cells were transfected with UHRF1-siRNA or EGFP-siRNA plasmid vectors and infected for the indicated times with *T. gondii* at a ratio of 1:1. UHRF1, cyclin B1 and actin proteins were then analyzed by Western blot. Data are representative of at least three independent experiments. **b** Binding of UHRF1 to *CCNB1* promoter in infected cells. BeWo cells were infected with *T. gondii* for the indicated times at a moi of 4:1. ChIP assay was performed using antibodies against UHRF1, or actin as negative control. Immunocomplexes were then analyzed by real-time PCR specific for the promoter region of *CCNB1*. Non-immunoprecipitated chromatin material was used as input control. The results were normalized to the input DNA and expressed as relative binding, compared to non-infected cells. Data are mean ± SEM of three separate experiments. ^*^P < 0.05, compared to non-infected cells

We have previously reported that UHRF1 is exploited by *T. gondii* to control *CCNB1* gene expression. In *T. gondii*-infected cells, increased levels of UHRF1 inversely correlate with levels of cyclin B1, leading to cell cycle dysregulation. mRNA levels of UHRF1 and cyclin B1 paralleled protein levels, meaning that these variations reflect changes in gene transcription [28]. To further establish whether UHRF1 is necessary and sufficient for *CCNB1* silencing, we transfected BeWo cells with UHRF1-siRNA plasmids in the presence or absence of *T. gondii* and evaluated cyclin B1 expression (Fig. 4a). Cells transfected with an EGFP-siRNA expression plasmid were used as controls. We observed a reversal of the infection-induced cyclin B1 kinetics in UHRF1 knock-down cells. A significant increase in cyclin B1 levels was observed at 12 h of infection in UHRF1 knock-down cells, compared with cells transfected with EGFP-siRNA, whereas lower expression is seen for earlier time points. These results indicate that UHRF1 is involved in cyclin B1 deregulation in *T.* *gondii*-infected cells.

To establish if UHRF1 is directly involved in cyclin B1 downregulation, we examined the recruitment of UHRF1 to the *CCNB1* promoter during infection, by chromatin immunoprecipitation (ChIP) assay (Fig. 4b). We observed enhanced UHRF1 binding to the *CCNB1* promoter at 3 h of infection. Control PCRs using primers for *GAPDH* cDNA demonstrated the specificity of UHRF1 precipitation (Suppl. Fig. 4). Together, these results show that, during *T.* *gondii* infection, UHRF1 negatively regulates cyclin B1 expression through recruitment on the *CCNB1* promoter.

### UHRF1 interacts with a multienzymatic complex on the *cyclin B1* promoter to induce histone modifications

**Fig. 5:**
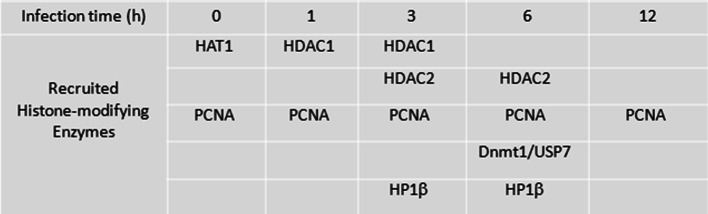
UHRF1 recruits a multienzymatic complex in *T. gondii*-infected cells. BeWo cells were infected with *T. gondii* for the indicated times at a ratio of 1:1. Whole protein cell lysates were separated by SDS-PAGE. Gel slices were cut in a systematic way and proteins were reduced, alkylated, and digested. The resulting peptides were analyzed by nanoLC-MS/MS. Proteins were identified through database searching, using a mixed human and toxo decoy database with a 1% false discovery rate. MS/MS spectra of the seven proteins of interest were extracted and manually validated to confirm the identifications

Following our previous finding that *T.* *gondii* downregulates cyclin B1 expression through UHRF1 activation and considering the established role of UHRF1 in epigenetic modification, we hypothesized that it might regulate cyclin B1 expression by interfering with the epigenetic machinery. UHRF1 is part of a multienzymatic chromatin-modifying complex [37]. To further characterize these UHRF1 containing immune complexes in *T.* *gondii*-infected cells, we immunoprecipitated UHRF1 at different time points and identified binding partners by mass spectrometry-based proteomics. SDS-PAGE gel of cell lysates, stained by Coomassie blue is presented in supplementary data (Suppl. Fig. 5). Altogether, 474, 564, 1063, 512 and 399 proteins were identified at *t* = 0 h, *t* = 1 h, *t* = 3 h, *t* = 6 h and *t* = 12 h, respectively (Suppl. Fig. 6). Seven proteins implicated in cell cycle control were identified at different time points (Fig. 5). All binding UHRF1 partners were validated by immunoprecipitation (data not shown). As shown in Fig. 5, we observed time-dependent interactions with the epigenetic-related proteins HDAC1 and HDAC2, PCNA, DNMT1, USP7, and HP1β.

**Fig. 6:**
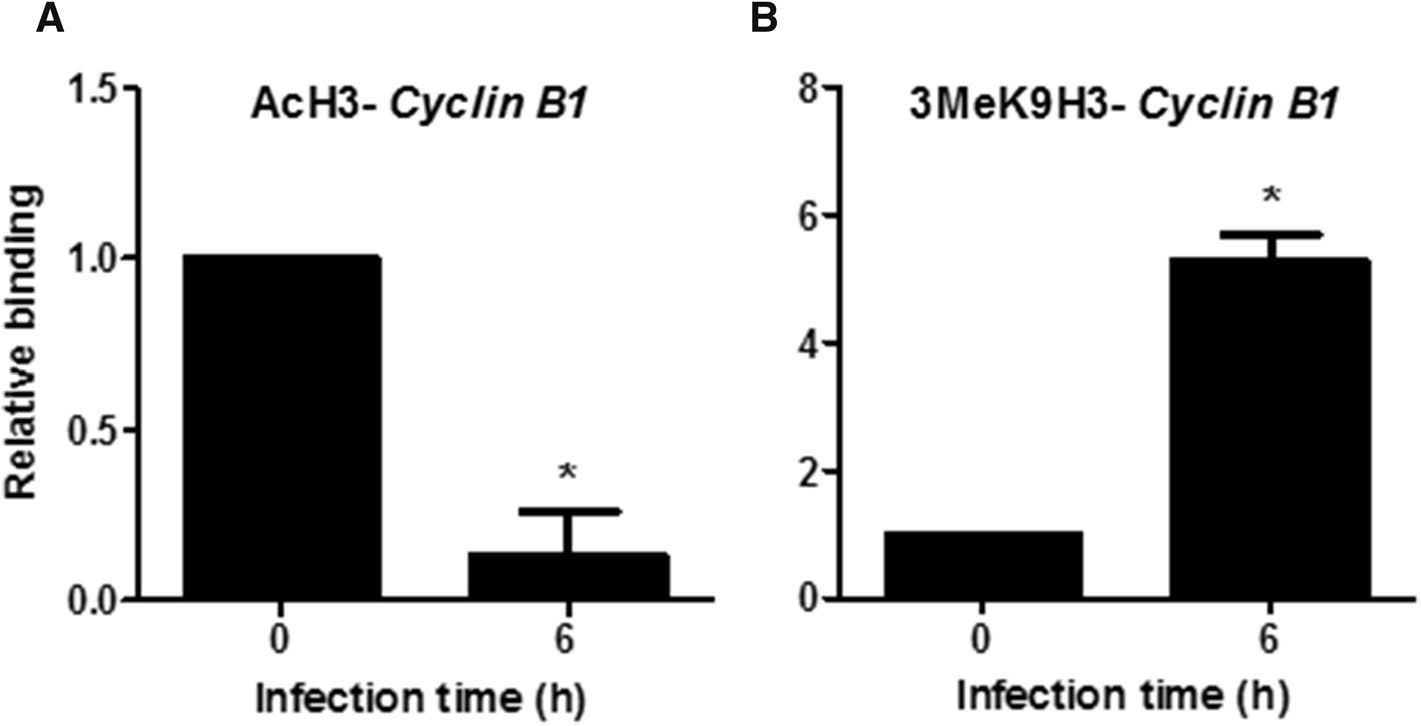
*T. gondii* interferes with histone H3 acetylation and methylation on *cyclin B1* promoter. BeWo cells were infected for the indicated times with *T. gondii* at a moi of 1:1. ChIP assay was performed using antibodies against acetyl-histone 3 **a** and trimethyl-H3 (Lys9) **b** or an isotype control antibody. The precipitated DNA was then amplified by real-time PCR for *CCNB1* promoter region. The results were normalized to the input DNA and expressed as relative binding, compared to non-infected cells. Data are mean ± SEM of three separate experiments. ^*^P < 0.05, compared to non-infected cells

We hypothesized that these interactions with histone deacetylases and methyltransferases might affect the histone marks on the *CCNB1* promoter in the course of infection. To clarify these time-dependent changes, ChIP assays were performed using antibodies against acetyl-H3 and H3K9me3, followed by *CCNB1*-specific PCR. We found a decrease of acetyl-H3 on the *CCNB1* promoter at 6 h post-infection (Fig. 6a), paralleled by an increase of H3K9 trimethylation (Fig. 6b). These results indicate that HDAC and methyltransferases, recruited with UHRF1 to the *CCNB1* promoter after infection, induce specific changes in histone acetylation and methylation levels.

### *T. gondii* activates DNMT expression in infected cells and regulates gene expression via DNA methylation

**Fig. 7:**
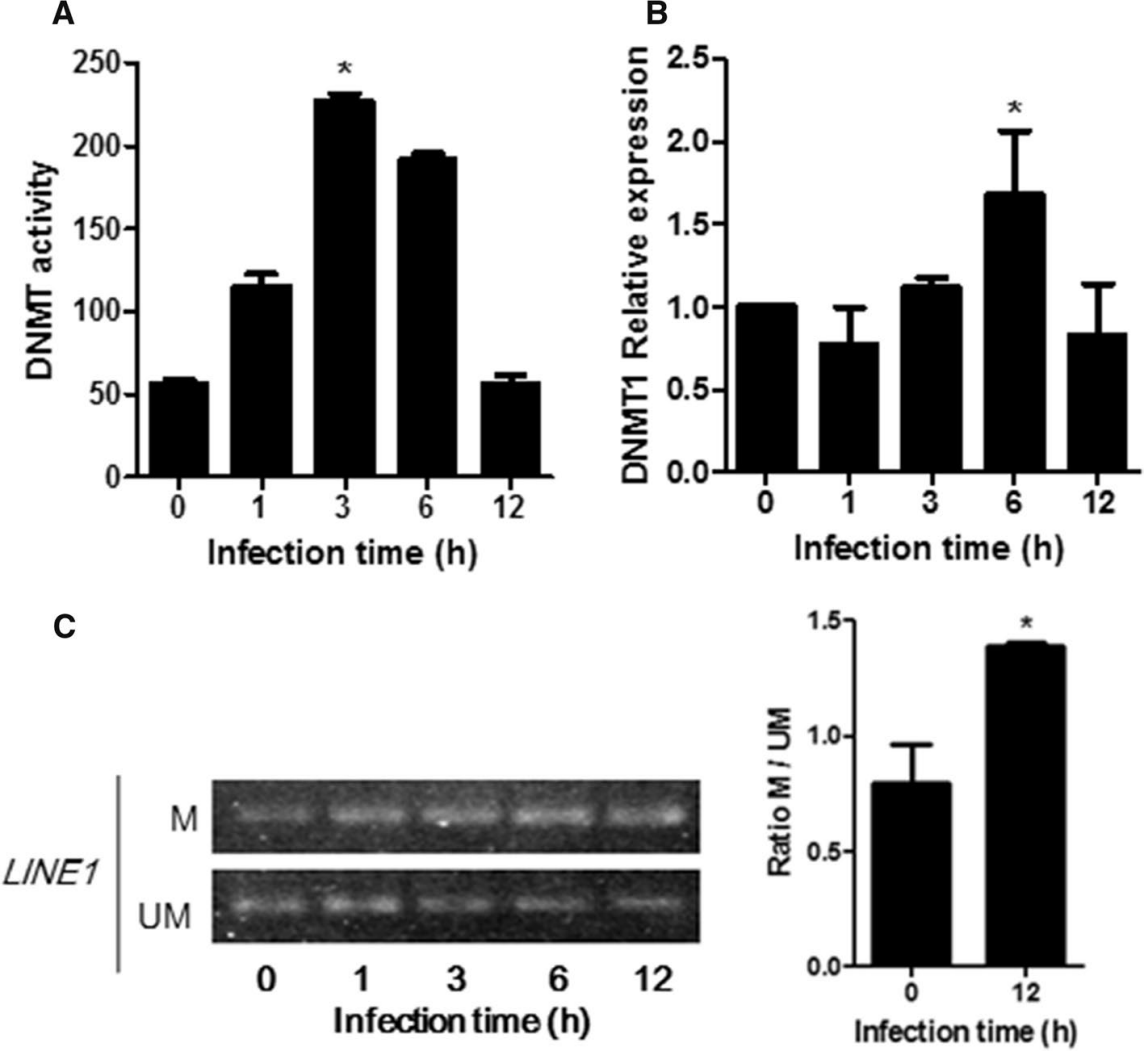
*T. gondii* induces DNMT expression and methylation activity. **a** The global DNMT activity during infection was analyzed by an ELISA-based method, as detailed in the methods section. **b** Cells were infected for the indicated times with *T. gondii* at a ratio of 1:1. DNMT1 mRNA levels were quantified by RT-PCR and presented relative to GAPDH expression. **c** BeWo cells were infected with *T. gondii* at. DNA was extracted and subjected to bisulfite treatment. Methylation of the *LINE1* sequence was assessed by PCR specific for methylated (M) or unmethylated (UM) sequences. Data are mean ± SEM of three separate experiments. ^*^P < 0.05, compared to non-infected cells

To get insight into the potential mechanisms of altered methylation status in infected cells, we determined the expression levels of the different DNMTs in *T. gondii*-infected cells. DNMT1 maintains the methylation pattern during replication, while DNMT3a and DNMT3b are de novo methyltransferases [38]. Our previous results led us to hypothesize that *T.* *gondii* could modulate DNMT activity to epigenetically silence the expression of target genes. We examined global DNMT activity in infected cells (Fig. 7a). Indeed, a significantly increased DNMT activity was evident as early as 3 h of infection. Quantitative RT-PCR analysis revealed that expression levels of DNMT1 were increased by parasite infection (Fig. 7b). In addition, to examine the *T.* *gondii* infection global methylation status, we performed a methylation-sensitive PCR of LINE sequences which are dispersed throughout the genome. As shown in Fig. 7c, methylated LINE sequences increased with infection, while unmethylated sequences decreased at the same time. Together, these results support our hypothesis that *T.* *gondii* infection induces hypermethylation of host genome DNA by increasing mediation by DNMT1 mRNA, protein levels and the global DNMT activity.

### *T. gondii* ROP16 kinase affects cyclin B1 expression and host cell cycle modulation

**Fig. 8:**
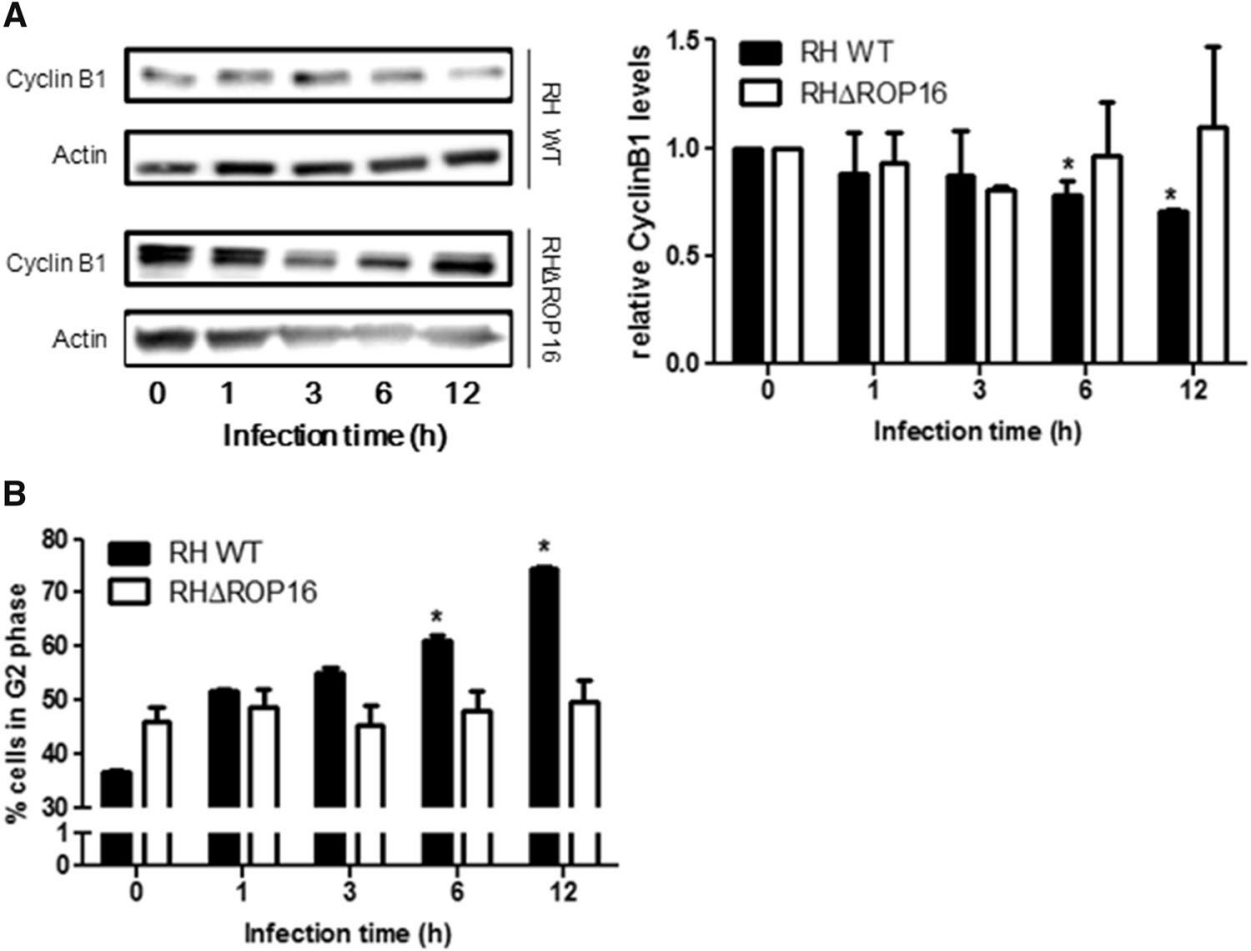
ROP16 plays a central role in UHRF1-dependant cyclin B1 regulation. **a** BeWo cells were infected for the indicated times with *T. gondii* RH WT or RHΔROP16 strains at a ratio of 1:1. Samples were analyzed by Western blot using antibodies against cyclin B1 or actin as loading control. Bands of three blots were analyzed by densitometry. **b** BeWo infected with *T. gondii* RH WT or RHΔROP16 strains were analyzed for cell cycle progression by flow cytometry of propidium iodide. Data are mean ± SEM of three separate experiments ^*^P < 0.05, compared to non-infected cells

Our previous results show an UHRF1-mediated regulation of *T. gondii*-infected cell cycle. To analyze the role of ROP16 in this process, we infected cells with RH WT and RHΔROP16 strains, and analyzed cyclin B1 expression levels and cell cycle (Fig. 8a, b). Infection with RHΔROP16 parasites did not result in decreased cyclin B1 expression, in contrast to the RH WT strain. Moreover, no augmentation of cells in G2/M phase was observed when infected with RHΔROP16 *Toxoplasma* strain, again in contrast to the RH WT strain. Percentages of cells in the different cell cycle phases during RHΔROP16 infection are shown in Suppl. Figure 7. Together, these results indicate that cyclin B1 and UHRF1-dependant cell cycle regulation in infected cells are ROP16 dependent.

## Discussion

The cell cycle is a target for intracellular microorganisms. For example, in HIV infection, cell cycle dysregulation is associated with enhanced virus proliferation [39]. We and others demonstrated such mechanisms with *T.* *gondii* infection, which induces an inhibition of cellular proliferation through arrest of the host cell cycle in the G2 phase via UHRF1 activation [28, 40]. The benefit of this modulation for the parasite remains unknown. Previous studies indicated an optimized nutrient acquisition or a compromised mitosis by parasite-induced rearrangement of host cell microtubules and recruitment of the microtubule organizing center to the parasitophorous vacuole membrane [41]. We previously showed that the cell cycle arrest is linked to a downregulation of *CCNB1* gene expression, associated with an overexpression of UHRF1 [28]. However, the parasitic factors inducing UHRF1 activation as well as the mechanisms of cell cycle regulation were unknown. Some authors hypothesized interactions between parasite proteins that cross the parasitophorous vacuole membrane and cyclin/CDK complexes that may affect their catalytic activities [40]. Recently, overexpression of the parasite kinase ROP16 was shown to be involved in Ser15/37 phosphorylation of p53, partial apoptosis of SH-SY5Y cells and cell cycle arrest in G1 stage [37]. We show here that ROP16 is one of the factors responsible for the UHRF1 activation, which in turn causes the down-regulation of cyclin B1 and the G2 cell cycle arrest. We demonstrate that activated UHRF1 is recruited to the *CCNB1* promoter during infection and induces epigenetic modifications, resulting in the formation of a heterochromatin environment and repression of the *CCNB1* promoter activity. This is the first report demonstrating that a parasitic infection, via its kinase ROP16, activates a transcription factor, UHRF1, leading to an epigenetic regulation process.

UHRF1 is a multi-domain-containing protein involved in epigenetic regulation through DNA methylation, histone deacetylation and methylation, and likely histone ubiquitination [23, 24]. To determine with which partners UHRF1 interacts with after *T.* *gondii* infection, we analyzed the enzymatic complexes associated with UHRF1 by mass spectrometry. Our results in infected cells show that UHRF1 interacts with HDAC1 and 2, and DNMT1 to form a repressive complex. Recruitment of this enzymatic complex leads to deacetylation of histone H3 and methylation of histone H3K9 on the *CCNB1* promoter, and consequently to its silencing. Besides histone modification, we also show that *T.* *gondii* infection causes DNA hypermethylation in the host cell by upregulation of DNMTs. H3K9 methylation and DNA methylation are well known to contribute to gene repression [37]. While this mechanism has never been described in a parasite infection model, in sporadic breast cancer, UHRF1 was shown to induce DNA methylation, as well as histone deacetylation and methylation on the *BRCA1* promoter by recruitment of an inhibitory transcriptional complex similar to the one we observed here [42]. The SRA domain of UHRF1 directly interacts with 5-hydroxymethylcytosine and hemi-methylated DNA and PCNA, which helps to recruit DNMT1 and stimulate its enzymatic activity [22, 43]. The TTD/PHD domains interact with methylated H3K9 which stimulates the enzymatic activity of H3K9 methyltransferases (H3K9MT) and thus methylation of adjacent H3K9, resulting in heterochromatin formation and gene silencing [24, 44].

During our mass spectrometry-based proteomics, we also show that UHRF1 interacts with USP7 in infected cells. USP7 (HAUSP) is a deubiquitylase that regulates DNMT1 and UHRF1 stability [45]. It is also targeted by *T.* *gondii* GRA16, binding PP2A-B55 to modulate genes involved in metabolism, cell cycle progression, and p53 tumor suppressor pathway [15]. Indeed, USP7 mediates deubiquitylation of UHRF1, preventing its proteasomal degradation. Moreover, UHRF1 is released from USP7 at the M phase of the cell cycle after its phosphorylation by CDK1-cyclin B1, leading to UHRF1 degradation [46]. Together with our results, this underlines once more the importance of cell cycle dysregulation in infected cells for maintaining a sufficient level of UHRF1 activity for parasite proliferation.

**Fig. 9:**
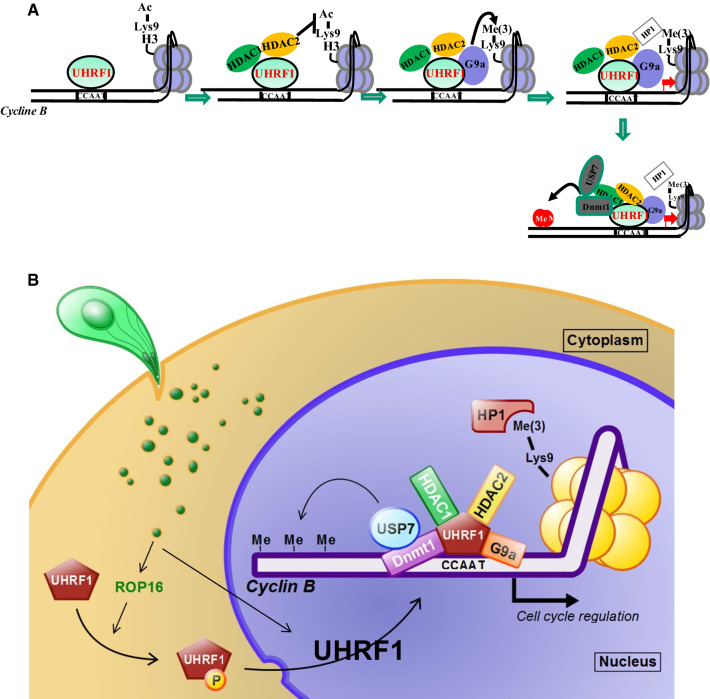
Model of UHRF1-mediated *cyclin B1* repression in *T. gondii*-infected cells. **a** In infected cells, the transcription factor UHRF1 binds to the *CCNB1* promoter where it recruits the histone deacetylases HDAC1 and 2 to promote local deacetylation of histone H3. UHRF1 also recruits histone methyltransferases which induce dimethylation of histone H3 allowing the recruitment of heterochromatin protein 1 (HP1β). Finally, the DNA methyltransferase DNMT1 is recruited inducing DNA methylation on the *CCNB1* promoter and thus heterochromatin formation and *CCNB1* gene silencing. **b** Model of *Toxoplasma*-induced regulation of cyclin B1 expression

Precise molecular mechanisms of UHRF1 domain interaction with the *CCNB1* promoter in parasite-dependent epigenetic events still need to be explored. However, based on our data, a global modeling of this interaction can be proposed (Fig. 9a). *T.* *gondii* modulates *CCNB1* gene expression by interfering with methylation and histone modifications. The recruitment of UHRF1 to the *CCNB1* promoter induces deacetylation of histone H3, by recruitment of HDAC 1 and 2. UHRF1 also recruits histone lysine methyltransferases which methylate the histone H3 and allow HP1β binding and heterochromatin formation. Finally, DNMTs are recruited by UHRF1 to methylate and silence the *CCNB1* promoter, resulting in cell cycle arrest in G2 phase. There is a similar example of the co-repressor COUP-TF interacting protein 2 (CTIP2) inhibiting *HIV*-*1* gene transcription by recruiting a chromatin-modifying complex and by establishing a heterochromatic environment at the *HIV*-*1* promoter in microglial cells, leading to HIV-1 silencing [47].

ROP16 is a polymorphic *T. gondii* protein kinase, targeting the host cell nucleus and known to directly induce phosphorylation of STAT3 and STAT6 [9, 13]. We show here that ROP16 also targets UHRF1. Our results suggest that UHRF1 is phosphorylated by ROP16 as an early event in cell invasion. Yamamoto et al. demonstrated that substitution of a single amino acid at position 503 (at the serine–threonine kinase domain) of the ROP16 protein in RH and Me49 strains determined a change in their activation of STAT3 [34]. Confirming the before mentioned findings for STAT3, we show that only the virulent isotype of ROP16 activates the UHRF1 gene promoter. This could be one clue to explain the mechanisms behind the striking differences on cytokine induction and parasite control between ROP16 isotypes in our recent in vivo study [11]. Preliminary work on PRU (type II) parasites, which contain the avirulent ROP16 isotype showed a graduate, but much less pronounced upregulation of UHRF1, while the cell cycle shows a considerably delayed (12–24 h) and less pronounced block, compared to type I infection (our own observation). This indicates that other factors besides ROP16 might interfere with host cell cycle, but they are likely to use other pathways and are obviously less effective. The in silico profile of the ROP16 protein of different strains should be explored, as Yamamoto et al. [13] had found that the residue at position 503 (leucine) in the RH strain is completely inaccessible to the substrate and that the cavity was larger for type II Me49 strain making the site more active.

Our results lead us to hypothesize that ROP16 injected early in infected cells phosphorylates UHRF1. In turn, phosphorylated UHRF1 translocates to the host nucleus and recruits the epigenetic complex leading to *CCNB1* promoter silencing and the observed cell cycle arrest in G2/M (Fig. 9b). As indicated by the lesser, but still visible increase of phosphorylated UHRF1 during RHΔROP16 infection, other p53-related mechanisms may be involved in the fine tuning of the cell cycle. Regulation in early stages of invasion through ROP16 or later through the secretion of proteins such as GRA16, GRA24 or MYR1 could maintain the non-replicative status of the cell [15, 29, 48].

Pathogen-induced alterations of host cell physiology aim to maximize its survival. Histone modifications and chromatin remodeling regulating gene expression should be key targets for intracellular parasites. In bacteria- or virus-induced host gene reprogramming, targets are the MAPK, IFN and NF-κB signaling pathways [49]. Well known in cancer development [24, 42], but unknown in host–pathogen interactions, we show the role played by UHRF1 during *Toxoplasma* parasitic infection.

In summary, we demonstrate in our model of trophoblastic cells that *T.* *gondii* ROP16 kinase activates UHRF1, which in turn regulates *CCNB1* epigenetic silencing through its recruitment on the *CCNB1* promoter and correlates with cell cycle dysregulation in these cells. Our data suggest that *CCNB1* epigenetic silencing is coordinated by UHRF1 through both DNA and histone methylation. Adding to the known transcription factors activated during infection, NF-κB, c-Fos, EGR1, c-Myc, STAT3/6, HIF1-α or NFAT4A, we can now include UHRF1, which emerges as an important epigenetic initiator regulating gene expression in *T.* *gondii*-infected cells along with NF-κB, p53, c-Myc STAT and HIF1-α [17]. Most likely, *CCNB1* is not the only gene targeted by UHRF1 and regulated in an epigenetic manner in infected cells, and further studies should broaden our understanding on this central transcription factor for *T. gondii* infection.

## Electronic supplementary material

Below is the link to the electronic supplementary material.

Supplementary material 1 (DOCX 14 kb)

Supplementary material 2 (TIFF 26 kb)

Supplementary material 3 (TIFF 88 kb)

Supplementary material 4 (TIFF 18 kb)

Supplementary material 5 (TIFF 49 kb)

Supplementary material 6 (TIFF 363 kb)

Supplementary material 7 (XLS 871 kb)

Supplementary material 8 (TIFF 40 kb)
