# Supplementary material for: Toxoplasma gondii ROP16 kinase silences the cyclin B1 gene promoter by hijacking host cell UHRF1-dependent epigenetic pathways
Source: Cell Mol Life Sci. 2019 Sep 6;77(11):2141–56. doi: 10.1007/s00018-019-03267-2 (PMC7256068; doi:10.1007/s00018-019-03267-2)
Supplement: Supplementary file 1 — Supplementary material 1 (DOCX 14 kb) [file 18_2019_3267_MOESM1_ESM.docx]

**Supplemental Information**

**Supplementary Figure 1:** ***UHRF1* promoter activity in non-infected BeWo cells.** BeWo cells were transfected with *UHRF1*-promoter luciferase reporter plasmid for 24 hours and assessed for luciferase activity (means ± SEM of three separate experiments performed in triplicate). * *P* < 0.05, compared to non-infected cells.

**Supplementary Figure 2:** ***T. gondii* infection activates *UHRF1* promoter and induces a cell cycle shift also in astrocytes, but less pronounced.** Astrocytes were transfected with *UHRF1*-promoter luciferase reporter plasmid for 24 hours, then infected for the indicated times with *T. gondii* (RH strain) at a moi of 1:1 and assessed for luciferase activity (A) and cell cycle progression (B). Values are shown as means ± SEM of three separate experiments performed in triplicate. * *P* < 0.05; ** *P* < 0.01, compared to non-infected cells.

**Supplementary Figure 3: *T. gondii* type I ROP16 is responsible for UHRF1** **activation in astrocytes.** Astrocytes were transfected with *UHRF1*-promoter luciferase reporter plasmid for 24 hours, then with ROP16 WT, ROP16 mutant plasmid without the catalytic kinase domain (ROP16ΔCat) (both from *T. gondii* RH strain) or the empty control plasmid. Luciferase activity was then analyzed and shown as means ± SEM of three separate experiments performed in triplicate. * *P* < 0.05, compared to control.

**Supplementary Figure 4: Binding of UHRF1 to *GAPDH* DNA in infected cells.** BeWo cells were infected with *T. gondii* for the indicated times at a moi of 4:1. ChIP assay was performed using antibodies against UHRF1. Immunocomplexes were then analyzed by PCR specific for *GAPDH* DNA. Non-immunoprecipitated chromatin material was used as input control.

**Supplementary Figure 5: Coomassie-stained gel for nanoLC-MS/MS analysis.** Non-infected (0h) BeWo cells or infected with *T. gondii* cells for the indicated times. Protein cell lysates were separated by SDS-PAGE and revealed by Coomassie blue.

**Supplementary Figure 6: Proteins identified by nano-LC-MS/MS-analysis.** Proteins recovered from BeWo cells after t=0h, 1h, 3h, 6h and 12h of infection with *T. gondii* were analyzed as described in Materials and Methods. Protein names, accession numbers and identified peptides in the individual experiments are shown for each time point. The identification table summarizes the findings for our proteins of interest, concerning cell cycle regulation.

**Supplementary Figure 7: Effect of infection with the *T. gondii* RHΔROP16 strain on cell cycle profile**. BeWo cells were infected with the *T. gondii* RHΔROP16 strain for the indicated times at a moi of 1:1. Uninfected control or T. gondii-infected cell population were analyzed by flow cytometry. The percentage of cells in each cell cycle phase of uninfected control cells or T. gondii-infected cell population were analyzed using propidium iodide at the indicated infection times.
