# Supplementary figures and images for: Toxoplasma gondii ROP16 kinase silences the cyclin B1 gene promoter by hijacking host cell UHRF1-dependent epigenetic pathways
Source: Cell Mol Life Sci. 2019 Sep 6;77(11):2141–56. doi: 10.1007/s00018-019-03267-2 (PMC7256068; doi:10.1007/s00018-019-03267-2)

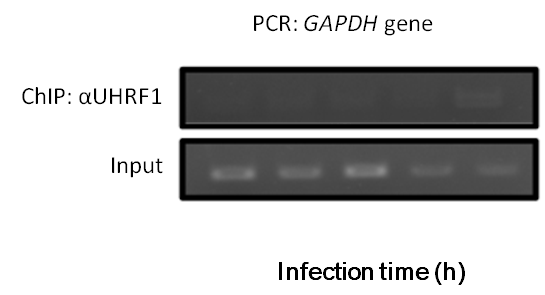

Supplement: Supplementary file 5 — Supplementary material 5 (TIFF 49 kb) [file 18_2019_3267_MOESM5_ESM.tif]

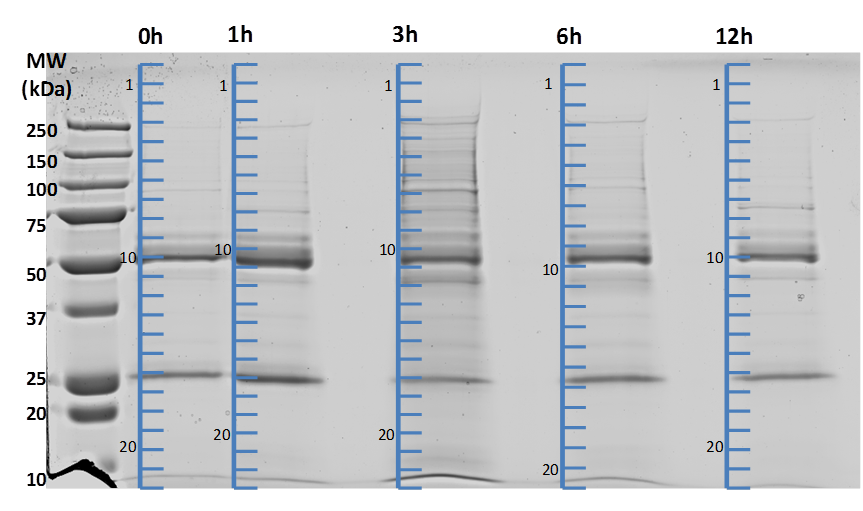

Supplement: Supplementary file 6 — Supplementary material 6 (TIFF 363 kb) [file 18_2019_3267_MOESM6_ESM.tif]
